# Transcatheter aortic valve implantation with a mechanically expandable prosthesis: a learning experience for permanent pacemaker implantation rate reduction

**DOI:** 10.1186/s40001-018-0310-4

**Published:** 2018-03-05

**Authors:** Jasmin Ortak, Giuseppe D’Ancona, Hüseyin Ince, Hüseyin U. Agma, Erdal Safak, Alper Öner, Stephan Kische

**Affiliations:** 1 Department of Cardiology, Vivantes Klinikum im Friedrichshain und Am Urban, Landsberger Allee 49, 10249 Berlin, Germany; 2 Rostock University Medical Center, Rostock, Germany

**Keywords:** Pacemaker, Aortic stenosis, Transcatheter, Aortic, Prosthesis

## Abstract

**Background:**

Permanent pacemaker implantation (PPMI) after transcatheter aortic valve implantation (TAVI) remains an issue open for criticism. Aim of this study is to investigate a strategy to reduce PPMI rate after TAVI in general and more specifically after implantation of the LOTUS^®^ prosthesis.

**Methods:**

Through our learning curve, we have developed a structured protocol to reduce PPMI rate. The protocol includes: shallow implantation depth within the native annulus, strict adherence to the international guidelines for PPMI, PPMI not earlier than 5 days after TAVI, and intravenous chronotropic and steroidal treatment (orciprenaline 0.6–1.7 mg/h i.v. and dexamethasone 25 mg/day i.v. for a maximum of 5 days) in case of acute onset of intraventricular and/or atrio-ventricular conduction disturbances after TAVI.

**Results:**

The first 35 patients (group A) were managed as per routine in our early stage experience with the LOTUS valve. The PPMI reduction protocol was applied in the second phase on the last 31 patients (group B). The PPMI rate was reduced from 34.3% (12/35) to 9.7% (3/31) (*p* = 0.02). At logistic regression analysis being treated in the second phase of our experience (group B) had a protective effect against PPMI (*p* = 0.05; OR = 0.1; CI = 0.01–1.0). Prosthesis implantation depth was directly related to PPMI (*p* = 0.005; OR = 2.0; CI = 1.2–3.2). Receiver operating characteristic curve analysis confirmed that a LOTUS implantation depth > 4.8 mm was the cut-off to predict PPMI (AUC = 0.8; *p* = 0.003; CI = 0.6–0.9) with maximal sensitivity (78.6%) and specificity (73.2%).

**Conclusions:**

PPMI rate after LOTUS can be reduced with experience by applying specific clinical and operative strategies.

## Background

Requirement for permanent pacemaker implantation (PPMI) after transcatheter aortic valve implantation (TAVI) remains an active unresolved issue. Its rate has been reported from 2 to 51% [1] with variations across studies and valve type.

In this context, in spite of its added feature of complete repositionability, which guarantees for optimization of final prosthesis position, the LOTUS^®^ valve (Boston Scientific, Marlborough, Massachusetts) has been exposed to criticism and restrictions in its application, in light of its increased requirement for post-procedural PPMI.

In the REPRISE II multicentre prospective trial, almost a third of the patients treated with the LOTUS prosthesis required post-procedural PPMI [2].

In the present manuscript, we present our single-centre experience with the LOTUS prosthesis and focus on the results achieved after having introduced a specific clinical protocol to limit the post-procedural PPMI rate.

## Methods

### Data collection

Patients undergoing trans-femoral TAVI with new generation LOTUS prosthesis were included. All patients in this series had been diagnosed with severe symptomatic A-V stenosis. Patients with the following conditions were excluded from the present analysis: pure aortic insufficiency, previous-AV surgery (replacement/repair), previous TAVI, and previous PPMI. Moreover, we have excluded from this series two patients where TAVI with the LOTUS was abandoned in favor of TAVI with a prosthesis of different model and larger size (one patient with 27.8 mm aortic annulus) or to convert to conventional aortic valve replacement (one patient developing left ventricular perforation during TAVI with LOTUS). Patients included in the present series were treated in the period 2014–2016 by our TAVI team.

Perioperative data were prospectively collected and reported according to the valve academic research consortium definitions (VARC).

### Procedure

All procedures were performed under fluoroscopic and trans-esophageal monitoring. Vascular access was percutaneous and through the right femoral artery in the majority of patients. Since the beginning of our experience, we have not performed aortic balloon valvuloplasty when implanting the LOTUS valve. Prosthesis selection has been routinely based upon preoperative evaluation of cardiac computed tomography images reconstructed using both the OSIRIX (Pixmeo SARL, Bernex, CH) and 3mensio (Pie Medical Imaging, Maastricht, NL) software. Since the beginning of our experience with the Lotus valve, we have adopted a policy of maximum 10% oversizing of the prosthesis in reference to the native annulus area-derived diameter.

After probing the native aortic valve, a pre-shaped stiff guidewire was used to advance the Lotus prosthesis. Details concerning the design and implantation of the Lotus prostheses have been previously described in the literature [2]. No patient received the most recent version of the Lotus prosthesis (i.e., Lotus Edge).

At the end of each procedure, prosthesis implantation depth was measured at the level of the non-coronary and left coronary cusps starting from the control aortography, in the nadir view. Measurements were obtained off-line after the procedure by an investigator that did not take part in the valve implantation.

### PPMI reduction protocol

Through our experience with the LOTUS valve, we have designed a structured protocol to reduce PPMI rate. After being reviewed by our internal research/ethics committee, and being submitted to the Berlin Medical Council, the protocol was implemented in the second phase of our experience with the Lotus (group B) and it is nowadays part of our standard operating procedure for TAVI.

**Fig. 1:**
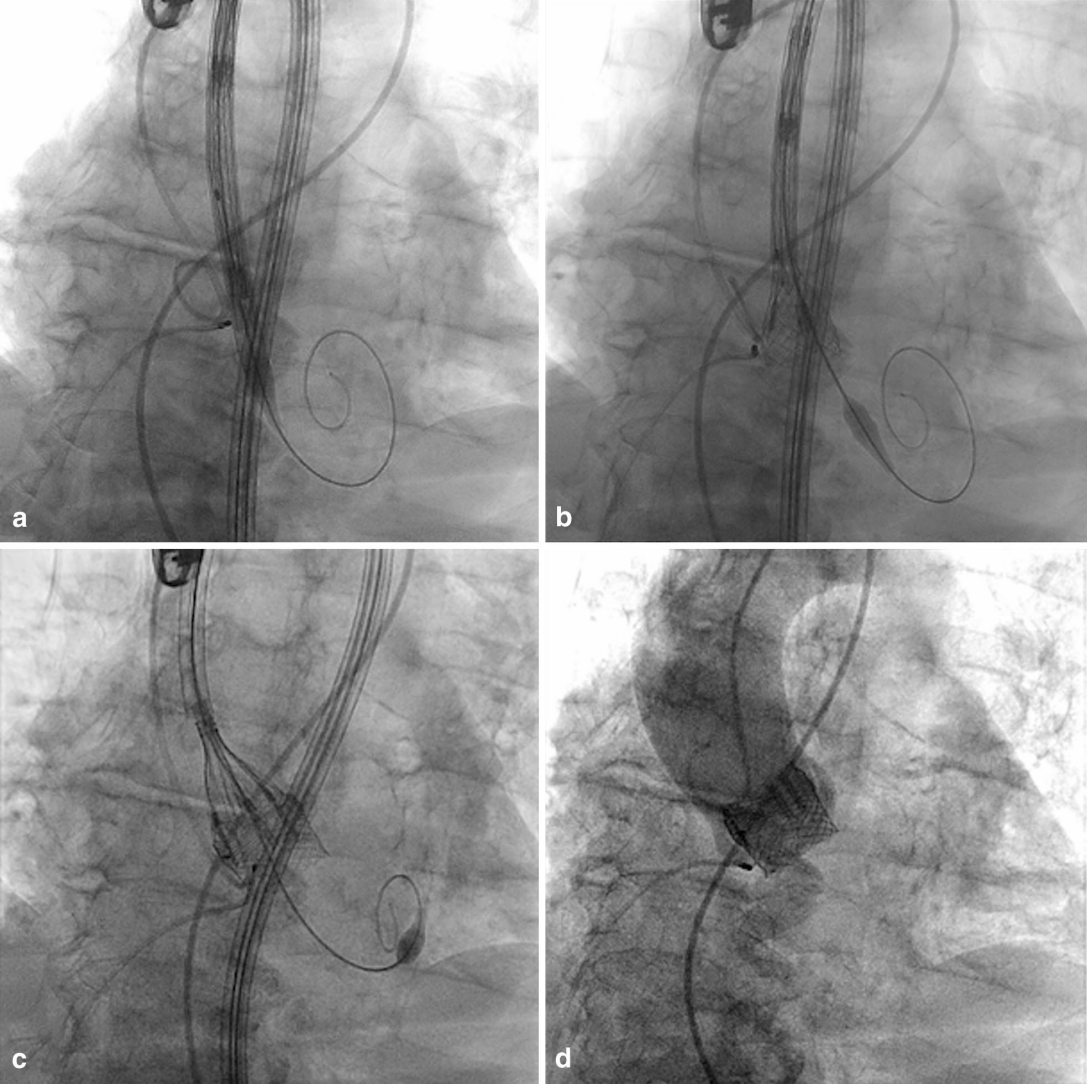
LOTUS implantation technique. **a** Initial position of LOTUS delivery system with marker pigtail placed in non-coronary cusp (nadir point) and distal end of valve nitinol frame depicted in projection of the annular plane. **b** Early stage of valve unsheathing with the valve nitinol frame reaching annular contact. Continuous backward tension on the delivery system helps maintaining a “high position” of the frame in respect to the native annulus. **c** The LOTUS nitinol frame starts to shorten and progressive “lay-over” of the delivery system with the main axis of the native aorta is achieved by applying forward pressure. **d** Final angiography demonstrating high annular position of the LOTUS valve with perfect annular sealing
- During the perioperative phases, a temporary PM is implanted from the jugular approach and left in place till the end of the procedure and for the first postoperative day. Postoperatively, the requirement for PPMI is based on the general policy of waiting until at least the 5th postoperative day after TAVI. In any case, the indications for PPMI are those proposed in the existing guidelines (Class I and II) and do not include new onset of intraventricular block such as left bundle branch block (LBBB) [3]. All patients are kept in telemetry for 120 h after TAVI. Moreover, a daily 12-lead EKG is performed and analyzed. If ECG findings that may require PPMI do not occur within 24 h after TAVI, the temporary PM is removed. In patients developing new ECG findings that, according to guidelines will require PPMI [3], the temporary PM is kept in place and activated in the demand mode at a rate of 50/min for a maximum of 120 h after TAVI. In the meantime, ECG is monitored daily. If ECG findings resolve and remain absent for at least 24 h, the temporary PM is removed. If ECG findings persist, PPMI is performed from the 5th postoperative day onward. Patient remains in an intensive care facility till the temporary PM is removed.
- Intravenous chronotropic agent (orciprenaline 0.6–1.7 mg/h i.v.) is started in case of new onset of second- or third-degree atrio-ventricular (A-V) conduction block. Steroidal treatment (dexamethasone 25 mg/day i.v.) is also associated. Both treatments are protracted until resolution of the ECG findings for a maximum of 5 days after TAVI. In patients with new onset of LBBB with a duration of > 120 ms, steroidal treatment alone is started (dexamethasone 25 mg/day i.v.) and protracted for a maximum of 5 days after TAVI. Dexamethasone use is refrained in case of recent history of gastrointestinal bleeding and documented severe immunosuppression.

- The protocol includes a modification of the original LOTUS implantation technique, as proposed by the manufacturing company, so to minimize direct peri-procedural trauma on the cardiac conduction system. From the very early phases of the implantation, the manufacturer suggests using as reference point the radiopaque marker of the prosthesis located in the middle of the valve nitinol frame. According to the manufacturer, the marker should be kept at the nadir of the native aortic annulus and this position should be corrected as the prosthesis position changes during the slow unsheathing of the device.
  Following these indications, the prosthesis will end up laying, during the distinct phases of valve positioning, deeper within the annulus and LVOT with at least half of its frame below the nadir of the native annulus.
  In our modified implantation technique, the nadir of the valve metallic frame is used as reference point and should be kept, at any given time during the procedure, immediately below the annular plane (not deeper than 5 mm) using a pigtail as reference in the deepest point of the non-coronary sinus (Fig. 1a–d). Once the valve is fully unsheathed and the frame has reached contact with the calcified annulus at the desired depth, a continuous and controlled pushing force is exerted on the prosthesis catheter. This is done to maintain the valve in position avoiding further shifting of the inferior edge of the valve nitinol frame above the annulus plane, while the valve progressively shortens to reach its final configuration.

### Statistical analysis

Data are presented as absolute numbers, percentages, mean ± standard deviation for normally distributed variables and median with minimum and maximum values for variables with not-normal distribution. Patients that did and did not require PPMI after TAVI as well as patients being treated before (group A) and after the introduction of our PPMI reduction protocol (group B) were compared. Differences of preoperative clinical/anatomical variables and perioperative management (including the PPMI reduction protocol) were analyzed. Paired Student *t* test, Wilcoxon signed-rank test, *χ*^2^, and Fischer-exact test were used whenever appropriate. A *p*-value < 0.05 was considered as significant.

A multivariable model was then built and stepwise backward logistic regression was performed to identify independent determinants for PPMI. Starting from the comparison between patients requiring and not requiring PPMI after TAVI with the LOTUS, we used in the logistic regression model only those variables that at univariate analysis had a statistical difference with a *p* < 0.05.

## Results

The first 35 patients (group A) were part of our initial experience with the LOTUS valve and were managed as per routine, following implantation directions proposed by the manufacturing company and presented in the existing literature. The post-procedural PPMI requirement in this group was 34.3% (12/35). At that stage, we anticipated a possible decrease of the observed PPMI rate by at least two-thirds (from 34.3 to 11.4%) when applying our PPMI rate reduction protocol. In this context, we calculated that the minimum number of subjects to be included in the PPMI reduction protocol for adequate study power was 27 (*α*: 0.05; *β*: 80%) [4].

We finally enrolled 31 patients in the PPMI reduction protocol (group B) and we reported a PPMI rate of 9.7% (3/31), confirming a decrease of more than two-thirds of the previously observed rate of 34.2% (*p* = 0.02).

Table 1 summarizes pre-procedural and peri-procedural data in the two groups.

**Table 1:** Preoperative and perioperative results in patients managed routinely (group A, early phase) or following a PPMI reduction protocol (group B, late phase)

	Group A (35)	Group B (31)	*p*-value
Age (years)	81.4 ± 6.1	79.4 ± 4.7	0.1
Female gender	57.1%	58.1%	0.9
Body mass index	27.3 ± 4.4	27.3 ± 4.2	0.9
LVEF%	55.6 ± 12.1	50.8 ± 16.1	0.1
A-V block first degree	8.6%	9.7%	0.6
Right bundle branch block	14.3%	12.9%	0.5
Left bundle branch block	20.0%	16.1%	0.4
Logistic Euro-score	5.1–49.7; 12.6	3.1–68.0; 14.2	0.2
Euro-score II	1.2–16.4; 3.5	2.0–28.0; 3.8	0.08
STS-score (mortality)	2.0–9.2; 3.5	2.0–16.0; 4.0	0.04
Annulus (mm)	24.4 ± 2.0	24.0 ± 1.9	0.4
LVOT (mm)	24.2 ± 2.1	24.0 ± 2.3	0.4
Bulbus aortae (mm)	31.0 ± 3.5	30.3 ± 2.2	0.4
Calcification vertical extension (mm)^a^	11.1 ± 4.8	11.3 ± 3.3	0.8
Operative time (min)	46–290; 78	40–135; 80	0.3
Radiation time (mm)	12–43; 23	11–64; 21	0.6
Valve retrieval	3%	9.6%	0.2
Valve full resheating	8.6%	19.2%	0.2
Valve post-dilatation	0	0	
Prosthesis size (mm)	24.9 ± 1.6	24.9 ± 1.5	0.9
Prosthesis/annulus	1.02 ± 0.06	1.03 ± 0.05	0.4
Aortic valve area (cm^2^)	1.5 ± 0.3	1.5 ± 0.4	0.8
Mean gradient (mmHg)	9.0 ± 4.5	6.3 ± 2.6	0.01
Paravalvular leak (0–1–2)	77.1%–22.9%–0%	87.9%–12.1%–0%	0.08
In-hospital death	0	9.6%	0.09
Stroke	0	0	
AKI	3%	3.8%	0.3
Bleeding (major–minor–none)	8.6%–23%–69%	0–26.9%–73.1%	0.3
New PPMI	34.3%	9.7%	0.02
Early safety (30-days)	100%	90.4%	0.09

LVOT, left ventricular outflow tract; AKI, acute kidney injury; PPMI, permanent pacemaker implantation. Data are presented as absolute numbers, percentages, mean ± standard deviation for normally distributed variables and median with minimum and maximum values for variables with not-normal distribution

^a^From deepest point of calcification to highest point of calcification measured at pre-operative cardiac CT (nadir–zenith)

A significantly higher risk profile was observed in group B that presented on average a higher STS-score (Table 1). The anatomical profile of the aortic unit was similar in the two groups, including a similar extent and distribution of aortic calcification (Table 1). Pre-procedural ECG findings were also similar in the two groups, including the presence of A-V and intraventricular conduction delays.

No significant differences were reported in peri-procedural findings (Table 1). In-hospital mortality and morbidity were similar in the two groups as well as technical success rates (Table 1). In-hospital/30-day mortality was 0% in group A and 9.7% in group B (*p* = 0.09). Of the three deceased patients in group B, none died as consequence of the procedure and/or the PPMI reduction protocol. In fact, one patient with depressed LVEF% (15%) and history of lymphoma (treated with radiotherapy 2 years before) died 20 days after TAVI for congestive heart failure leading to multi-organ failure. A second patient with history of diverticulosis died for intestinal perforation leading to peritonitis 10 days after TAVI. A third patient, with chronic obstructive pulmonary disease (FEV1 30% of predicted value and oxygen therapy), developed complete A-V block, acute pulmonary failure followed by pneumonia and sepsis that led to death 10 days after TAVI. None of the three deceased patients in group B had received post-procedural dexamethasone treatment.

In total new LBBB after TAVI were recorded in 37.1% of patients in group A and 32.3% of patients in group B (*p* = 0.4), new complete A-V blocks in 28 and 11.9% (one in group B resolved) (*p* = 0.1), and first degree A-V conduction delay in 8.6 and 12.9% (*p* = 0.4).

All patients requiring PPMI in group A were implanted within 48 h from TAVI. In two cases in group A (16% of total PPMI in group A) PPMI was guided by new onset of intraventricular conduction delay (LBBB) with progressively widening QRS complex at serial ECG controls (> 150 ms after 48 h). The remaining 10 patients (83% of total PPMI in group A) were implanted for new onset of complete third-degree A-V block that did not improve within 48 h after TAVI. Concerning group B, two out of three patients requiring PPMI were implanted 5 days post-TAVI, after being submitted to an unsuccessful treatment with orciprenaline and dexamethasone. The third patient was never implanted with PPM as result of lethal acute on chronic respiratory failure, pneumonia, and septic shock that occurred 4 days after TAVI and led to death 10 days after TAVI (see mortality above). This patient never received dexamethasone treatment being already managed with high-dose steroids in light of his severe COPD.

In any case, all three patients are accounted for in our PPMI calculation for group B. In fact, they all had a constant and clear indication for PPMI with complete third-degree A-V block.

In group B, one patient with pre-operative atrial fibrillation developed a new LBBB and intermittent third-degree A-V block that were resolved after 5 days of treatment with dexamethasone and orciprenaline and, for this reason, he was discharged without PPMI on day 14 after TAVI. In three cases, a > 120 ms post-procedural LBBB was resolved after initiation of dexamethasone treatment.

In synthesis, in group B a total of four patients (12.9%) required i.v. orciprenaline and six patients (19.3%) i.v. dexamethasone. None of these patients experienced in-hospital death.

To build an adequate model for PPMI prediction, we compared patients requiring and not requiring PPMI after TAVI with the LOTUS valve. Table 2 summarizes univariate comparison of preoperative and perioperative findings. Only three variables were significantly different in the two groups (with and without PPMI).

**Table 2:** Preoperative and perioperative results in patients requiring and not requiring PPMI after TAVI with the LOTUS prosthesis

	With PPMI (15)	Without PPMI (51)	*p*-value
Age (years)	82.4 ± 5.7	79.9 ± 5.5	0.1
Female gender	46.7%	60.8%	0.3
Body mass index	27.1 ± 5.1	27.4 ± 4.1	0.8
LVEF%	57.0 ± 8.8	52.5 ± 15.0	0.2
Atrial fibrillation	26.7%	19.6%	0.4
A-V block first degree	20.0%	6.0%	0.1
Right bundle branch block	26.7%	9.8%	0.1
Left bundle branch block	6.7%	21.6%	0.1
Logistic Euro-score	5.0–49.7; 12.4	3.1–68.0; 14.0	0.5
Euro-score II	1.2–16.4; 3.4	1.6–27.9; 3.5	0.4
STS-score	1.0–9.1; 3.3	1.2–16.6; 3.7	0.4
Annulus (mm)	24.5 ± 1.4	24.1 ± 2.1	0.5
LVOT (mm)	24.2 ± 1.6	24.1 ± 2.3	0.9
Bulbus aortae (mm)	31.6 ± 3.1	30.5 ± 3.1	0.2
Calcification vertical extension (mm)^a^	9.4 ± 3.3	11.7 ± 4.4	0.2
Operative time (min)	60–180; 78	40–290; 80	0.4
Radiation time (mm)	14.5–64.7; 24	11.1–42.5; 21.6	0.1
Group B	20%	55%	0.02
Valve retrieval	20%	2.2%	0.04
Valve full resheating	13.3%	13.0%	0.1
Prosthesis size (mm)	25.5 ± 1.5	24.7 ± 1.5	0.09
Prosthesis/annulus	1.05 ± 0.05	1.02 ± 0.06	0.2
Aortic valve area (cm^2^)	1.7 ± 0.6	1.5 ± 0.3	0.1
Mean gradient (mmHg)	7.5 ± 2.8	7.8 ± 4.3	0.7
Paravalvular leak (0–1–2)	86.7%–13.3%–0	80.4%–19.6%–0	0.7
In-hospital death	6.7%	3.9%	0.5
Hospitalization length (days)	12.2 ± 8.5	8.1 ± 4.7	0.02
Implantation depth mm (left coronary cusp)	5.7 ± 1.8	3.4 ± 1.9	< 0.0001
Implantation depth mm (non-coronary cusp)	3.5 ± 2.9	3.4 ± 2.1	0.9

LVOT, left ventricular outflow tract. Data are presented as absolute numbers, percentages, mean ± standard deviation for normally distributed variables and median with minimum and maximum values for variables with not-normal distribution

^a^From deepest point of calcification to highest point of calcification measured at pre-operative cardiac CT (nadir–zenith)

Patients requiring PPMI were less often belonging to group B (*p* = 0.02), had a significantly deeper LOTUS implantation depth (*p* < 0.0001), and had undergone more frequently a full valve retrieval to implant a LOTUS of a larger size (*p* = 0.04) (Table 2).

At logistic regression analysis only two variables were independently related to PPMI. Belonging to group B had a protective effect against post-TAVI PPMI (*p* = 0.05; OR = 0.1; CI = 0.01–1.0) and prosthesis implantation depth was directly related to post-TAVI PPMI (*p* = 0.005; OR = 2.0; CI = 1.2–3.2). Receiver operating characteristic (ROC) curve analysis was performed to better identify the relationship between prosthesis implantation depth and PPMI. A nitinol frame depth of 4.8 mm at the level of the left coronary sinus was the cut-off to predict PPMI after TAVI with the LOTUS prosthesis (AUC = 0.8; *p* = 0.003; CI = 0.6–0.9) with maximal sensitivity (78.6%) and specificity (73.2%) (positive predictive value 25.3% and negative predictive value 91.5%).

### Follow-up

All surviving patients underwent at least one follow-up visit, including surface ECG analysis, within 1 month from discharge.

No new PPMI was necessary. Out of the 14 patients implanted with PPM, nine (64%) were PM-dependent at follow-up (eight in group A and one in group B).

**Fig. 2:**
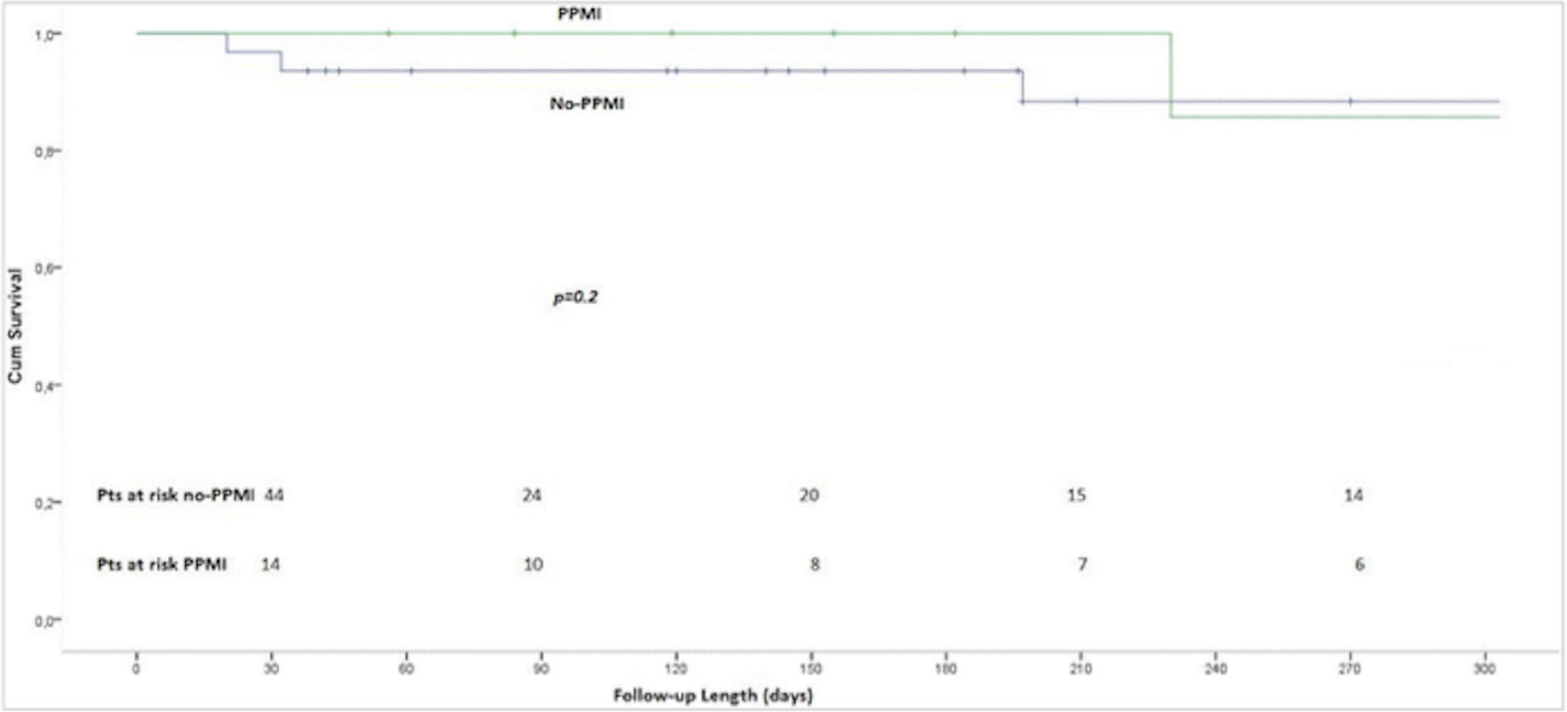
Survival curves after TAVI with the LOTUS valve. Survival curves in patients with and without new PPMI after TAVI with the LOTUS prosthesis

At a median clinical follow-up of 209 days (30–721 days), estimated survival in patients with PPMI after TAVI was 85.7 and 88.7% in those that did not require PPMI (*p* = 0.3) (Fig. 2).

None of the surviving patients required a new PPMI and none developed complications resulting from the perioperative management.

## Discussion

Aortic valve stenosis and regurgitation are associated, per se, with cardiac conduction delays, including high degrees of A-V block [5, 6]. In fact, histologic abnormalities of the conducting system, together with fibrous thickening of the endocardium of the ventricular septum, are often present in patients with aortic valve disease and may have mechanical (increased left ventricular pressures) and ischemic etiology [7].

In the REPRISE II trial, a 30-day and 1-year PPMI rate ranging from 28.6 to 31.9% was reported in 120 patients, after TAVI with the LOTUS valve [2, 8]. Similar results have been proposed in the RESPOND post-market study that includes over 500 patients treated with the LOTUS prosthesis (presented at PCR London Valves 2015).

We believe that in general PPMI rate after TAVI may result from a combination of device-specific features, implantation technique, and post-procedural management, including a very “preventive” strategy for PPMI after TAVI.

### LOTUS features

The LOTUS TAVI prosthesis is mechanically expandable and presents some peculiar features. Due to its elevated radial force, this prosthesis results in high rates of full expansion. At post-procedural computed tomography evaluation, the LOTUS prosthesis shows almost complete circularization of the native annulus that results in low trans-prosthetic gradients and minimal paravalvular leak rates [9]. Its particular radial strength, exerted within the native annulus, may also lead to an increased compression on the bordering anatomical structures, such as the cardiac conduction tissues. It has to be emphasized that the very last design of the Lotus valve (Lotus Edge), that presents an improved profile of catheter and prosthesis to diminish left ventricular outflow tract traumatism, was not employed in the presented series.

### Valve sizing, implantation technique, and prosthesis depth

Since the beginning of our experience with the Lotus prosthesis, we have adopted a concept of minimal prosthesis oversizing, to reduce the trauma exerted on the landing zone. As a result, the prosthesis/annulus size ratio is similar in the two temporal cohorts. As documented in our results, PPMI may be necessary even in patients treated with minimal valve oversizing. This is possibly due to the prosthesis implantation technique and to the final position of the valve, at time of release. In fact, the relationship between implantation depth and PPMI rate has been shown since the beginning of the TAVI experience with the Medtronic Core-Valve System (Medtronic Inc., Minneapolis, Minnesota) [10] and it has been restated in more recent studies [11]. Very recently, Zaman et al. have confirmed in a series of 95 Lotus patients, a PPMI rate of 28% [12]. When chasing for independent predictors of PPMI, pre-existing RBBB [hazard ratio (HR) 2.8, 95% CI 1.1–7.0; *p*  =  0.032] and depth of implantation below the non-coronary cusp (HR 2.4, 95% CI 1.0–5.7; *p*  =  0.045) were the strongest determinants [12].

It is reasonable to think that, apart from the final position of the valve within the annulus, the intermediate phases before achieving final valve release may also impact upon PPMI rate. In fact, trauma to the conduction tissue can happen at any time during the valve release process and result from a direct injury exerted by the valve frame and/or valve catheter. This is of particular importance when adopting a mechanically expandable prosthesis with elevated radial force such as the LOTUS prosthesis. In light of these considerations, the core of our proposed LOTUS implantation strategy is to minimize, at any given point during the procedure, impingement of the prosthesis and catheter too deep into the left ventricular outflow tract. To reduce this eventuality, we have tried to monitor the valve depth within the aortic annulus during the various phases going from valve advancement into the left ventricular outflow tract, valve unsheathing, complete expansion, and shortening. As already emphasized in the methods section, we propose an implantation technique where the nadir of the valve metallic frame should be kept, at any given time during the procedure, immediately below the annular plane (approximately 5–10 mm) using a pigtail as reference in the deepest point of the non-coronary sinus. To achieve this goal, we have also observed an increment in the valve re-sheathing rate. The re-sheathing was actually adopted to fine-tune and optimize the final position of the valve. Our implantation differs from that originally proposed by the manufacturing company and actually routinely applied in the first phase of our experience with the LOTUS. In fact, the manufacturer suggests implanting the valve using as reference point the radiopaque marker located in the middle of the valve nitinol frame. By following the manufacturer indication the prosthesis lays, during the various phases of valve positioning, deeper within the annulus and LVOT with at least half of its frame below the nadir of the native annulus. While the valve shortens and reaches its final position, it squeezes and pulls upward whatever tissue it has anchored during the earlier phases of positioning.

When looking more specifically at the prosthesis final position, it is now accepted that the shallower the implantation depth the lower the PPMI rate. Petronio et al. have confirmed, by means of receiver operating curve analysis, that a Core-valve implantation depth of < 4 mm at the level of the non-coronary cusp is a negative predictor of PPMI [11]. In fact, the bundle of His passes through the right fibrous trigone and the membranous ventricular septum and gives off fibers that form the left bundle beneath the commissure between the right and non-coronary cusps, in close vicinity to the aortic valve, over a distance of 6.5–20 mm [13].

The negative predictive values of PPMI according to prosthesis depth resulting from both ours and Petronio et al. analysis [11] support the fact that, when implanting the prosthesis shallower than a certain depth cut-off, the chances of not requiring a PPMI are around 90%. On the contrary, once the depth cut-off has been overcome and the valve is implanted deeper within the annulus, the probability of requiring a pacemaker is around 20% (positive predictive values of, respectively, 24.8 and 21.7%). This confirms that, apart from prosthesis final depth, there are many other clinical and perioperative factors that may impact upon PPMI rate.

In synthesis, our results substantiate the importance of implantation depth even when using the LOTUS device [12] and we were actually able to identify an ideal cut-off after which the risk of PPMI should increase. It should be noticed that in our analysis depth at the level of the left coronary sinus, instead of the non-coronary sinus, seemed to play a stronger and independent impact upon PPMI rate. In fact in our experience, in spite of the self-centering properties of the LOTUS prosthesis, once the valve is fully released the nadir of the nitinol frame is most often at the level of the left coronary cusp.

### Post-procedural management

We have structured part of our proposed PPMI reduction protocol, including dexamethasone and orciprenaline treatment, on the basis of previous findings in the field of percutaneous treatment of congenital heart disease. After percutaneous closure of perimembranous ventricular septal defects, cardiac conduction delays may result directly from mechanical compression, inflammation, and fibrosis. The septal occluder device has been shown to cause a localized inflammatory reaction that can result in extensive scar tissue and cartilaginous metaplasia of the surrounding myocardium [14]. Conduction abnormalities after TAVI may have a similar ground and the proposed protocol, although completely original in the TAVI field, has its own logic.

The use of steroids has been initially reported to be successful for treatment of atrio-ventricular block in inflammatory diseases [15]. More recently, at least two groups have proposed an empirical regimen of steroids, non-steroidal anti-inflammatories, and chronotropic agents to manage iatrogenic heart block after percutaneous closure of ventricular septal defects [16, 17].

Although our initial findings seem encouraging (with one patient experiencing regression of an intermittent complete A-V block and three patients showing regression of an initial LBBB > 130 ms), we do not have enough data to conclude that the PPMI rate was actually reduced thanks to the application of this pharmacological regimen. In fact, acquisition of confidence and dexterity with the Lotus valve was possibly the main factor that allowed us to reduce the PPMI rate as we gained experience. Moreover, we have to remark that in three cases complete A-V block did not improve after 5 days of treatment with positive chronotropic agents (in three patients) and steroids (in only two patients). Finally, the risks of high-dose steroidal treatment should be considered. In our experience, dexamethasone administration did not lead to major morbidity and/or mortality and we limited its use to patients developing complete A-V block and LBBB with widening QRS (> 120 ms). In any case at the present, we are testing the pharmacological protocol in a larger sample, including patients implanted with other valve types.

### Indications for PPMI and implantation timing

In the past, prophylactic PPMI has been proposed, and used, also in patients with new onset of LBBB and prolonged PR interval after TAVI, not in line with the international recommendations for PPMI [3]. The possibility of reducing PPMI rate after TAVI by strictly adhering to international guidelines for PPMI has been just recently proposed [3, 11, 18].

We have categorically started to follow the international guidelines for PPMI just in the second phase of our experience with the LOTUS valve (PPMI reduction protocol in group B). Using this more conservative approach, no total A-V block or sudden death occurred between discharge and follow-up, even in patients that had developed LBBB after TAVI. This is an important point because it is not yet clear how a widening QRS complex is going to evolve after implantation of a TAVI prosthesis with such high radial force.

Timing for PPMI should also be viewed critically to minimize PPMI rate. In both the REPRISE II trial and RESPOND post-market study, the majority of PPMs were implanted within 2 days from TAVI with LOTUS, and more than half within 24 h. Interestingly, less than 50% of patients undergoing PPMI were pacemaker-dependent after 30 days [2, 8].

These findings confirm the fact that injuries to the conduction system occurring during TAVI may be temporary, mainly related to localized edema and inflammation and consequently may heal in due time. A closer look to the surgical literature shows that in some centers it is policy waiting until at least the 5th postoperative day before performing PPMI after conventional aortic valve replacement [19]. To safely optimize the timing of PPMI after TAVI, we have revised our management of temporary pacemakers. Introducing the temporary PM via the jugular vein instead of the femoral vein may be advisable to reduce the infection risk and to allow for patient mobilization, whenever the PM is kept in place to wait for recovery of normal cardiac conduction. Although keeping a temporary lead in place for prolonged time may generate concerns, a more liberal approach towards PPMI will also add invasiveness and iatrogenic risks. In any case in our experience, a temporary PM was kept in place for up to 5 days after TAVI in six patients. Patients were kept on telemetry in a high-dependency unit and in none of them the temporary lead resulted in iatrogenic complications such as cardiac perforation and/or infection.

## Limitations

The PPMI reduction protocol adopted in the second phase of our experience with the LOTUS valve and presented in the present manuscript represents an evolution of our modus operandi and it is still under evaluation. The study design is not a prospective case–control study but a comparison of patients treated at two different time points (i.e., before and after the development of our PPMI reduction protocol) and, as an inevitable consequence, with different degrees of experience. For this reason, it is difficult to understand how much the different actions guided by the protocol may have impacted upon reduction of PPMI. In fact, PPMI reduction may be just resulting from an acquisition of experience in handling the prosthesis. In reality, the protocol we have proposed in the present manuscript is the result of our “learning curve” with the LOTUS device. Certain parts of the protocol, such as the use of positive chronotropic agents and steroids, are absolute novelties and are being tested as this manuscript is formulated. They may have a clinical logic but their routine use is not justified if not within the premises of registered studies.

## Conclusion

Requirement for PPMI after TAVI remains an issue. It is known that the occurrence of A-V conduction disturbances requiring post-procedural PPMI after TAVI could be related to many factors such as patients’ clinical and anatomical profile, implantation technique and implantation depth, valve design/sizing, and peri-procedural management, including indication to and timing for PPMI. We have proposed an innovative strategy to reduce PPMI after TAVI. Although the presented protocol may have a general application in TAVI patients, we have presented its results in patients who have undergone LOTUS valve implantation. The protocol includes important steps in the preoperative and perioperative patients’ management. Although our results in a small cohort are encouraging, they will need to be tested in the next future within the premises of larger studies.

## Abbreviations

PPMI: permanent pacemaker implantation
TAVI: transcatheter aortic valve implantation
ROC: receiver operating characteristic
AUC: area under curve
VARC: valve academic research consortium
LVEF: left ventricular ejection fraction
LBBB: left bundle branch block
RBBB: right bundle branch block
